# The complete chloroplast genome of *Pinus squamata* (Pinaceae), a critically endangered species in China

**DOI:** 10.1080/23802359.2019.1644224

**Published:** 2019-07-22

**Authors:** Li-Zhen Ling, Shu-Dong Zhang

**Affiliations:** School of Biological Sciences and Technology, Liupanshui Normal University, Liupanshui, China

**Keywords:** Chloroplast genome, phylogenetic analysis, *Pinus squamata*, *Pinus* subsect. Gerardianae

## Abstract

The complete chloroplast (cp) genome of *Pinus squamata*, a critically endangered species in China, was reported in this study. The cp genome is 118,300 bp in length and has four regions: 65,298 bp of large single copy (LSC) region and 52,098 bp of small single copy (SSC) region separated by 452 bp of highly reduced inverted repeat (IR) regions. This cp genome with GC content of 38.8% contains 107 unique genes (73 protein-coding genes, 30 tRNA genes, and four rRNA genes). Phylogenetic analysis based on the complete cp genomes indicates that *P. squamata* is closer to *P. gerardiana* than *P. bungeana*.

*Pinus squamata* X.W. Li is a fast-growing, straight-stemmed tree with a smooth off-white bark. It is a critically endangered species (Yang and Christian 2013) and only 32 wild individuals are found in the field (Zhang et al. 2005). This species is listed as a first-grade state protection plant of China. Low level of genetic diversity detected in *P. squamata* (Zhang and Li 2003; Zhang et al. 2005) might lead to an increased risk of extinction. To promote the conservation of this species, we sequenced and analyzed its complete chloroplast (cp) genome using high-throughput sequencing technology.

The voucher specimen (Wang et al. 03-0431) was collected from Yaoshan Mountain (Qiaojia, Yunnan, China; 103°00′E, 26°52′N) and deposited at Herbarium, Kunming Institute of Botany, CAS (KUN). Genomic DNA was extracted from the silica-gel-dried leaves and used for the library construction and Illumina sequencing. Approximately 6 Gb raw data were used for the cp genome assembly using the GetOrganelle pipeline (https://github.com/Kinggerm/GetOrganelle). The cp genome annotation was accomplished using Dual Organellar Genome Annotator (DOGMA) (Wyman et al. 2004) coupled with manual check and adjustment.

The complete cp genome of *P. squamata* (GenBank accession number: MK994519) is 118,300 bp in length with a typical quadripartite structure containing two highly reduced inverted repeats (IRs) of 452 bp, a large single copy (LSC) region of 65,298 bp and a small single copy (SSC) region of 52,098 bp. The overall GC content of cp genome is 38.8%. A total of 107 unique genes consist of 73 protein-coding genes, 30 transfer RNA (tRNA) genes, and four ribosomal RNA (rRNA) genes, which is similar to other species of *Pinus* (Yang et al. 2018). Among these genes, 12 genes (*atpF*, *petB*, *petD*, *rpl2*, *rpl16*, *rpoC1*, *trnA-UGC*, *trnG-UCC*, *trnI-GAU*, *trnK-UUU*, *trnL-UAA*, and *trnV-UAC*) possess a single intron and 2 genes (*rps12* and *ycf3*) have two introns.

*Pinus* subsect. *Gerardianae*, including *P. squamata*, *P. bungeana*, and *P. gerardiana*, is stable monophyly (Zhang et al. 2003). However, the phylogenetic relationships of three species are controversial. Based on eight plastid gene sequences (*matK*, *rbcL*, *trnV*, *ycf1*, *accD*, *rpl20*, *rpoB*, and *rpoC1*), *P. squamata* was sister to the clade formed by *P. gerardiana* and *P. bungeana* (Saladin et al. 2017). However, the phylogenetic tree with the nuclear ITS sequences supported *P. squamata* and *P. gerardiana* were sister to each other before clustering with *P. bungeana* (Zhang et al. 2003). To determine the phylogenetic position of *P. squamata*, phylogenetic analysis was performed with the complete cp genome of *P. squamata* and four previously released cp genomes of *P. gerardiana* and *P. bungeana*. *Pinus krempfii* was used as the outgroup. The phylogenomic trees generated through maximum-likelihood (ML) and Bayesian inference (BI) methods (Ronquist et al. 2012; Stamatakis 2014) were identical in topology (Figure 1). The trees supported *P. squamata* was most closely related to *P. gerardiana* (Figure 1), which was consistent with the phylogenetic study using ITS sequences (Zhang et al. 2003).

**Figure 1.**
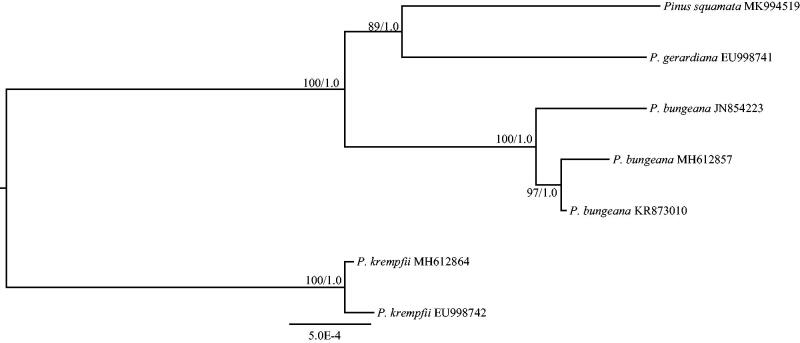
The maximum-likelihood (ML) tree of *Pinus* subsect. *Gerardianae* inferred from the complete chloroplast genome sequences. Numbers at nodes correspond to ML bootstrap percentages (1,000 replicates) and Bayesian inference (BI) posterior probabilities.
